# A retrospective audit of pain assessment and management post-caesarean section at New Somerset Hospital in Cape Town, South Africa

**DOI:** 10.4102/safp.v63i1.5320

**Published:** 2021-09-30

**Authors:** Effraim F. Munsaka, Dominique van Dyk, Romy Parker

**Affiliations:** 1 Department of Anaesthesia and Perioperative Medicine, Faculty of Health Sciences, University of Cape Town and Groote Schuur Hospital, Cape Town, South Africa

**Keywords:** pain assessment, pain management, post caesarean delivery, caesarean section, multimodal analgesia

## Abstract

**Background:**

The most common major surgical procedure performed worldwide is the caesarean section (CS). Effective pain management is a priority for women undergoing this procedure, to reduce the incidence of persistent pain (a risk factor for postpartum depression), as well as optimise maternal-neonatal bonding and the successful establishment of breastfeeding. Multimodal analgesia is the gold standard for post-CS analgesia. At present, no perioperative pain management protocols could be identified for the management of patients presenting for CS at regional hospitals in South Africa. This audit aimed to review the folders of patients who underwent CS, with particular reference to perioperative pain management guidelines for CS.

**Methods:**

A descriptive, retrospective, cross-sectional audit was conducted. Three hundred folders (10% of the annual number of caesarean procedures performed) from New Somerset Hospital, a regional hospital in Cape Town, South Africa were reviewed.

**Results:**

The women were a mean age of 30 years (standard deviation [s.d.]: 6.2). Median gravidity was 3 (interquartile range [IQR]: 2–3) and parity was 1 (IQR: 1–2); 52% had previously undergone a CS. In 93.3% cases, spinal anaesthesia was employed for CS. Pain assessment was poor, with only 55 (18%) patients having their pain assessed on the day of the operation. Analgesia was prescribed in over 98% of the patients, however, medication was only administered as prescribed in 32.6%. Non-steroidal anti-inflammatory drugs (NSAIDs) were prescribed in < 5% of cases. None of the patients received a patient-controlled analgesia (PCA), transversus abdominis plane (TAP) block, or wound infusion catheter as supplementary strategies.

**Conclusion:**

Pain management for post-CS patient at this hospital is lacking. There is the need for the implementation of a structured assessment tool to improve administration of analgesics in these patients. In addition, the reasons for the omission of NSAIDs from the analgesia regimen requires investigation. Hospital requires post-CS pain protocols to guide management especially in resource-limited settings.

## Introduction

The most common major surgical procedure performed worldwide is the caesarean section (CS).^1^ Effective pain management is a priority for women undergoing this procedure, to reduce the incidence of persistent pain (a risk factor for postpartum depression), as well as optimising maternal-neonatal bonding and the successful establishment of breastfeeding after delivery. The reported incidence of persistent incisional pain or the need for analgesia beyond 6 months after a CS varies markedly, between 1% and 18%.^2^ A prospective observational study at a regional hospital in Cape Town showed that the first 24 h after CS under spinal anaesthesia was the period with the highest incidence of moderate to severe pain (84%).^3^

Pain is a subjective phenomenon defined by the International Association for the Study of Pain as an ‘unpleasant sensory and emotional experience associated with actual, or resembling that associated with actual, or potential tissue damage’.^3,4,5^ As a fifth vital sign, pain should be routinely assessed, managed (if indicated), and re-assessed. Given the subjectivity of pain, the gold standard for its assessment is a validated self-reporting tool. The most commonly used tools for evaluating pain intensity include the Likert-type numeric rating and the visual analogue scales.^3,6,7^ Other pain assessment tools available include the Verbal Rating Scale (VRS), McGill Pain Questionnaire,^8^ Wong-Baker Faces Pain rating scale,^9^ and the Pain Quality Assessment Scale.^3^

The American Pain Society (APS) recommends that planning for post-operative pain management should begin in the preoperative period, and physicians should focus on individualising perioperative pain management using a multimodal approach.^10^ The 2016 Guidelines on the Management of Post-operative Pain,^11^ provide several recommendations relevant to this audit, including that: (1) Clinicians conduct a preoperative evaluation including the assessment of medical and psychiatric comorbidities, concomitant medications, a history of chronic pain, substance abuse, and previous post-operative treatment regimens and responses, to guide perioperative pain management plans; (2) Clinicians adjust pain management plans based on the adequacy of pain relief and the presence of adverse events; (3) Clinicians use a validated assessment tool to track responses to post-operative pain treatments and adjust the treatment plans accordingly; (4) Clinicians offer multimodal analgesia, or the use of a variety of analgesia medications and techniques combined with non-pharmacological interventions, for the treatment of post-operative pain in children and adults.

Multimodal analgesia is the gold standard approach for post-CS analgesia management.^12^ One strategy uses neuraxial morphine, scheduled non-steroidal anti-inflammatory drugs (NSAIDs), and paracetamol, and limits systemic opioids to the treatment of breakthrough pain.^13^ The South African Society of Anaesthesiologists (SASA) also recommends multimodal analgesia as the most effective way of alleviating acute pain post CS and that patients be discharged on oral pain medication and/or suppositories.^14^ Such analgesia options are appropriate for most parturients, but there are many women whose medical conditions require special consideration. Some conditions that will require alterations to pain management include: preeclampsia, side-effects to previously administered analgesic medications, pre-existing chronic pain, obstructive sleep apnoea, psychiatric comorbidities, and any contraindication to neuraxial anaesthesia.^10^

At present, no perioperative pain management protocols appear to be in existence for the management of patients presenting for CS at a regional hospital in South Africa. A protocol is defined as ‘a detailed written set of instructions to guide the care of a patient or to assist the practitioner in the performance of a procedure’.^15^ Protocols are useful tools to assist healthcare professionals translate guidelines into practice. Patients presenting to regional hospitals for CS would be expected to have a higher incidence of comorbidities than their counterparts in district-level hospitals, and healthcare professionals at these institutions potentially have access to a wider range of analgesic options. Therefore, regional hospitals’ specific protocols should include guideline-based information on the management of the higher risk patient presenting for CS.

Prior to making recommendations to develop and implement protocols, it is good practice to conduct an audit to describe current clinical practice. Clinical audits are used to improve patient care and evaluate outcomes as part of a continuous cycle essential in evidence-based medicine to optimise and update patient care.^16^ This audit aimed to review the folders of patients who underwent CS with particular reference to perioperative pain management guidelines for CS.

## Method

This audit was a query review^17^ which requires that at least 10% of cases or a minimum of 40 cases be reviewed. This study looked at patients who underwent CS over a 1-year period from 01 December 2017 to 31 November 2018. Approximately 220–250 CS are conducted per month at New Somerset Hospital, or 3000 per year. Therefore, 300 folders (10%) were reviewed. The principal author obtained the information from the theatre register for procedures done during that period and compiled a list of all patients who had undergone a CS using names and folder numbers. The list was then entered into a Microsoft Excel spreadsheet and their folder numbers were randomised using the inbuilt randomisation feature to generate a list of 300 random folders for the review.

### Measurements

**FIGURE 1:**
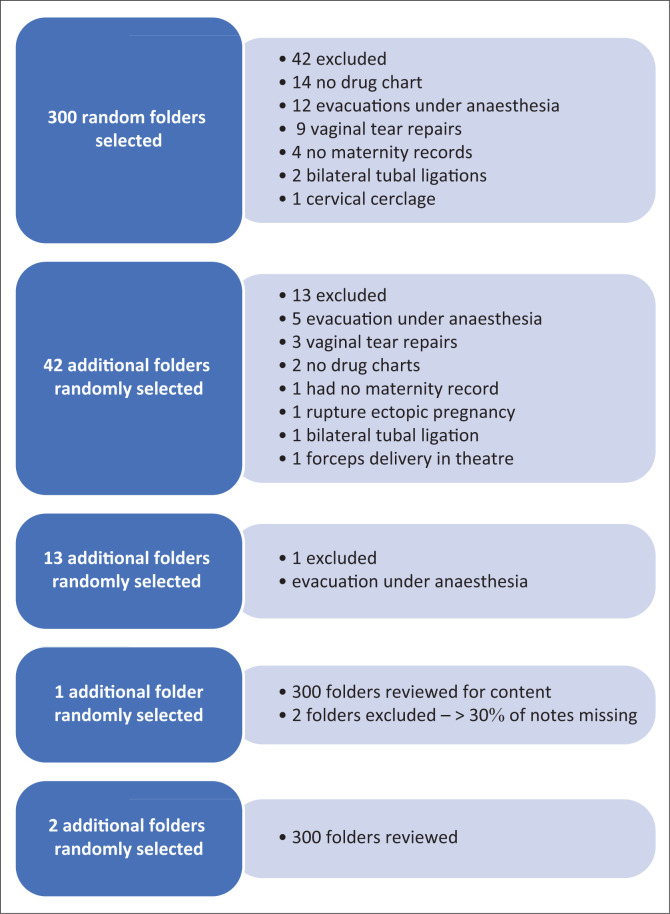
Flow chart of folder selection process to obtain 300 random folders.

The information obtained from the folders included sociodemographic and health information, management of the CS, and the modalities of post-operative pain assessment and management. The REDCap software (version 3.8.4) data collection tool was used to upload the information, with password protection. A pilot trial of 20 folders was done initially to test the usability of the tool and subsequently discarded. As illustrated in Figure 1, 358 folders were randomly selected but 58 folders were excluded because the surgical procedure was not a CS, thus the appropriate number were added to the list to make 300 in total.

### Ethical considerations

The work presented in this article was granted ethical approval by the University of Cape Town, Faculty of Health Sciences, Human Research Ethics Committee (HREC reference: 703/2018) and the Western Cape Government Health Research Department (reference: WC_201901_008).

## Results

### Sociodemographic history

As seen in Table 1, the women were a mean age of 30 (standard deviation [s.d.]: 6.2) years old. The most common comorbidities were human immunodeficiency virus (HIV) (18%); hypertension related to pregnancy (9%), and asthma (4%). Median gravidity was 3 (interquartile range [IQR]: 2–3) and parity was 1 (IQR: 1–2). Fifty-two percent had previously undergone a CS (median 1; IQR: 1–2).

**TABLE 1:** Sociodemographic and health profile of patients (*n* = 300).

Activity	Mean	(s.d.)	Median	range	IQR
Age (years)	29.96	6.20	-	-	-
**Comorbidities**					
Hypertensive disorders of pregnancy	27	9.00	-	-	-
Asthma	12	4.00	-	-	-
HIV	53	17.70	-	-	-
Epilepsy	4	1.30	-	-	-
Depression	3	1.00	-	-	-
Eczema	1	0.33	-	-	-
Syphilis	1	0.33	-	-	-
Avascular necrosis of the hip	1	0.33	-	-	-
**Past Obstetric History**					
Gravidity	-	-	3	1–9	2–3
Parity	-	-	1	0–6	1–2
Previously had a CS	-	-	-	-	-
Median number of CS (*n* = 157)	157	52.3	1	1–3	1–2
Previously experienced a miscarriage	90	30.00	-	-	-
Median number of miscarriages (*n* = 90)	-	-	1	1–6	1–1

s.d., standard deviation; IQR, interquartile range; CS, caesarean section; HIV, human immunodeficiency virus.

### Management of caesarean section

Spinal anaesthesia was used in 93.3% of the patients for the management of CS. Based on the folder review, common practice at this hospital during the study period was the intrathecal administration of 10 mg of 0.5% hyperbaric plus 10 mg of fentanyl. This was sometimes supplemented with one or more of the following agents: intravenous (IV) paracetamol, ketamine, and fentanyl. Patients who required general anaesthesia (GA) received a combination of morphine, IV paracetamol and fentanyl for pain relief. Other analgesic agents included ketamine, alfentanil and local infiltration with plain bupivacaine. However, only one patient received a wound infusion catheter while none had transversus abdominis plane (TAP) blocks employed. Details of intraoperative management appear in Table 2.

**TABLE 2:** Management of current caesarean section (*n* = 300).

Activity	*n*	%
**Indication for CS**		
Foetal	103	34.33
Foetal and maternal	77	25.67
Maternal	120	40.00
**Type of anaesthetic received**		
General	20	6.70
Spinal	280	93.30
**Intra-operative pain management**		
**Patients receiving GA (*n* = 20)**		
IV/IM morphine	16	80.00
IV paracetamol	17	85.00
IV ketamine	3	15.00
IV fentanyl	10	50.00
IV NSAIDs	0	0.00
IV alfentanil	7	35.00
Local anaesthesia infiltration (0.25% Plain Bupivacaine)	5	25.00
Wound infusion catheter	0	0.00
Peripheral nerve block	0	0.00
**Patients receiving Spinal (*n* = 280)**		
IV/IM morphine	6	2.14
IV paracetamol	20	7.14
IV ketamine	13	4.60
IV fentanyl	11	3.92
IV NSAIDs	4	1.42
IV alfentanil	0	0.00
Local anaesthesia infiltration (0.25% Plain Bupivacaine)	16	5.71
Wound infusion catheter	1	0.36
Peripheral nerve block	0	0.00

IM, intramuscular; IV, intravenous; NSAIDS, non-steroidal anti-inflammatory drugs; CS, caesarean section; GA, general anaesthesia.

The most common indications for CS were maternal (40.0%), namely previous CS, declining vaginal birth after CS (VBAC), and hypertensive disorders of pregnancy, especially preeclampsia. Other indications included: foetal (34.3%) and both foetal and maternal (25.6%). Of the 300 live births (280 spinal, 20 GA), 288 of the neonates went straight to their mothers following delivery, and 12 required Neonatal Intensive Care Unit (NICU) care (11 GA, 1 spinal anaesthetic).

### Post-operative pain management

#### Pain Assessment

Spinal anaesthesia was the most common modality used for the management of CS (93.3%). This technique should allow the opportunity for earlier and better establishment of pain control in the post-operative recovery area, as patients are wide-awake immediately after their procedures and regression of the spinal block can be assessed. A pain assessment was recorded as having been conducted based on the presence of any form of documented pain assessment in either the doctors’ or nurses’ notes, for example, ‘mild pain’ or ‘patient complaining of pain’. The VRS was the only scoring system used for the assessment of pain during the period under review. The rate of documented follow-up of the response to pain management was low, with 13 of the 55 patients who had their pain assessed on the day of surgery (day 1), having their pain reassessed after administration of analgesia (24%) (Table 3). On day 2, 21/33 (64%) of patients had their pain assessed, and were reassessed after administration of analgesia. On day 3, 13/30 (43%) of patients had their pain reassessed after administration of analgesia.

**TABLE 3:** Frequency and method of pain assessment and reassessment.

Activity	Day 1		Day 2		Day 3	
	*n*	%	*n*	%	*n*	%
**Pain Assessed (*n* = 300)**	55	18	33	11	30	10
**Method of assessment**						
VRS	17	31	26	79	21	70
Patient complained of pain	38	69	7	21	9	30
**Pain reassessed after administration of analgesia**	13	24	21	64	13	43
Consistent reassessment	2	0	3	0	2	0
Intermittent reassessment	11	0	18	0	11	0

VRS, Verbal Rating Scale.

Method of assessment day 1: *n* = 55; method of assessment day 2: *n* = 33; method of assessment day 3: *n* = 30.

#### Pain management

In the chart review, data was extracted on what analgesics were prescribed, and what analgesics were administered based on documented evidence of administration. Analgesia was prescribed in over 98% of the patients, with the medication administered as prescribed in 32.6% of patients on the first day and 37% on the second day (Table 4). Oral paracetamol and morphine were prescribed in 99.7% and 82.0% of cases respectively. Minimal use of oral NSAIDs was evidenced by low prescription rates throughout (4.0% – 5.0%). None of the patients received either patient-controlled analgesia (PCA) or TAP block, while only one patient received a wound infusion catheter (with local anaesthetic) as supplementary strategies. Details of prescription and administration of analgesia appear in Table 4.

**TABLE 4:** Prescription and administration of analgesia.

Activity	Day 1		Day 2		Day 3	
	*n*	%	*n*	%	*n*	%
**Analgesia prescribed**	299	99.67	300	100.00	296	98.67
**Analgesia received as prescribed**						
Yes	98	32.67	113	37.67	98	32.67
No	4	1.34	1	0.33	4	1.34
Intermittent	193	64.34	186	62.00	190	63.33
Declined	4	1.34	-	-	4	1.34
Medicine not obtained from pharmacy	1	0.33	-	-	1	0.33
**Type of analgesia prescribed**						
Oral paracetamol	299	99.67	298	99.33	293	97.67
Oral NSAID	5	1.67	4	1.33	4	1.33
IV/IM morphine	246	82.00	184	61.33	168	56.00
PCA	0	0.00	0	0.00	0	0.00
IV/IM pethidine	0	0.00	0	0.00	0	0.00

IV, intravenous; NSAID, non-steroidal anti-inflammatory drug; IV/IM, intravenous/intramuscular; PCA, patient-controlled analgesia.

## Discussion

This retrospective audit of 300 folders explored the documentation and implementation of pain assessment and management over a period of 1 year in women who had undergone CS at a regional hospital in Cape Town. The typical woman presenting to this hospital for this procedure was 30 years old and presenting for a second or third CS (52.3%). Our folder review showed consistency in the prescription of analgesia postoperatively with the use of more than one form of analgesic, mostly paracetamol and morphine. However, very few patients received NSAIDs, and no supplementary blocks, wound infusion catheters, or PCA devices were employed, such that the principle of multimodal analgesia was not followed. In addition, the prescribed medicines were not reliably administered. The subjective character of pain and the complexity of the feelings evoked by pain make reliable measurement by health professionals a key factor in successful management.^18^ However, limited documentation of pain assessment and reassessment was observed.^19^

The most used post-operative pain assessment tools are unidimensional and assess only pain intensity, which is just one aspect of the sensory dimension.^20^ These include the Numerical Rating Scale (NRS) and the VRS. The McGill Pain Questionnaire is one of the most frequently used multidimensional pain assessment tools^21^ and measures aspects of pain including the physical and emotional characteristics.^20^ In this study, the VRS was the only documented pain assessment method. According to Williamson,^22^ most patients prefer the VRS because it is easier to use compared to NRS and visual analogue scale (VAS) even though it lacks sensitivity and the data it captures can be misunderstood. On the day of surgery, the VRS was used in 31% of the folders. Since this is a retrospective chart review, it is possible that pain was assessed in more patients but was not documented. However, this lack of documented pain assessment provides a learning point for medical personnel and the development of a protocol. All pain should be regularly monitored and evaluated,^23^ management should be documented and followed up. Poor documentation hinders periodic appraisal of clinical practice and has potential medico-legal implications.^3,24^

Neuraxial anaesthesia techniques, specifically spinal anaesthesia, was the most commonly used method for CS in this chart review (93.3%). This is a strategy that is being adopted throughout the world as it has been associated with reduced rates of maternal mortality.^25^ In addition, it has been shown that patients undergoing CS under GA have a higher frequency of pain than patients receiving spinal anaesthesia.^26,27^ The most common practice at this hospital appeared to be intrathecal administration of 10 mg hyperbaric bupivacaine plus 10 mg fentanyl. Compared to GA, spinal anaesthesia provides for early assessment and engagement of the patient in the management of pain. In line with the SASA Acute Pain Guidelines,^14^ a patient has the right to be believed, to be properly assessed, to access appropriate effective pain management strategies, to be educated on the effective pain management options, and to be cared for by health professionals with training and experience in the management of pain. This approach provides for patient engagement and maintaining patient autonomy.

Multimodal analgesia should include scheduled NSAIDS and paracetamol with opioids reserved for severe breakthrough pain.^28^ The APS guidelines recommend that pharmacological agents should include a neuraxial opioid in conjunction with non-opioid adjuncts such as scheduled NSAIDs and paracetamol, with additional opioids reserved for severe breakthrough pain.^10^ It has been suggested that intrathecal morphine be a gold standard for post-caesarean pain as it provides excellent and prolonged post-operative analgesia.^28^ This folder review revealed that post-operatively, most patients received an analgesic regimen of mostly morphine and paracetamol. Of concern was a very low prescription rate for NSAIDs (4% – 5%), despite drugs like Ibuprofen being readily available as it is included in on the Standard Treatment Guidelines and Essential Medicines List for South Africa.^29^ There is documented evidence that NSAIDs have opioid-sparing effects, with a consequent reduction in opioid-related side effects.^30^ The combination of NSAIDs and morphine has been used extensively, and NSAIDs have been shown to reduce morphine use by 33% – 47%,^31^ when administered either as a single bolus or scheduled medication. Furthermore, the combined use of paracetamol with diclofenac resulted in a 38% reduction in the use of morphine, compared against patients receiving paracetamol only.^32^ Non-steroidal anti-inflammatory are mild-to-moderate analgesics which, when combined with paracetamol have synergistic anti-inflammatory properties and should form the backbone of pain management in these patients. The very low prescription rate for NSAIDs in this chart review despite numerous guidelines recommending their use is concerning. There is an urgent need to establish the reasons behind this finding.

Other analgesic modalities worth exploring include PCA, TAP blocks and wound infusion catheters. This review observed that none of the patients received PCA or TAP blocks, while only one patient had a wound infusion catheter (with local anaesthetic). This is not unusual in resource-limited settings were lack of adequate staffing, education, and post-operative monitoring facilities limit how much can be offered to a patient.^33^ Bilateral TAP blocks and wound infusion catheters,^33,34^ have been shown to reduce post-operative opioid consumption and nausea and vomiting, and are associated with lower post-operative pain scores. However, continuous wound infiltration via a catheter requires supplementary equipment (catheters, infusion pumps that may not be readily available),^33^ while a portable ultrasound machine is required to safely perform TAP blocks. Disposable PCA devices, on the other hand, have the advantages in that they do not require electricity or battery usage, but remain expensive and not readily available compared with foxed-dose opioid prescriptions, coupled with the high volume of obstetric cases.^33^

This review, as with many retrospective chart reviews, was fraught with many challenges. These highlight areas requiring further research, the need for training clinicians in better record keeping, pain evaluation and management, and the need to develop post-CS pain management protocols at this hospital. The South African Acute Pain (SAAP) guidelines recommend constituting a pain team and the need to document and evaluate.^14^ At this hospital, the practice appears to be that anaesthetists are only involved in the pain management of post-CS patients admitted to intensive care or high care unit. The development of a protocol with the relatively available drugs will provide the necessary guidance for institutions across the country where constituting such multidisciplinary teams may, for various reasons, be challenging.

Retrospective chart reviews are limited by convenience sampling, the inability to determine causation (only association), reliance upon the accuracy of written record, difficult to control bias and confounders,^35^ misclassification bias, and temporal relationships often difficult to assess. A future prospective study would be more appropriate for the capture of relevant information in real time including pain assessment tools used, the socioeconomic status of the women, cultural and/or behavioural perspectives on pain and its management, challenges in assessment and recording of pain management by health professionals, and ward follow-up of patients. However, based on these findings in this review, a pain management protocol for CS specific for the type of anaesthesia that has been applied, and which includes scheduled NSAIDs is needed. This protocol should be developed with clear delineation of responsibilities between anaesthetists, obstetricians and nurses, and then implemented ensuing a quality improvement cycle with re-evaluation every 3–6 months to ascertain its utility and effect on patient outcomes.

## Conclusion

Pain management is not merely about the reduction of pain; it is also about the optimisation of recovery through reliable and accurate assessment of pain.^3^ Effective post-operative pain management is imperative in increasing patient safety and satisfaction, and reducing costs to the healthcare services.^20,36^ It is concerning that according to this chart review, post-CS patients are not being assessed for pain nor receiving adequate pain management. A significant proportion of the pain interventions appeared to be based on the professional knowledge of the practitioner and are not supported by evidence-based guidelines of pain management.^11^ There is need for a post-CS nurse-led pain management protocol which specifies: (1) the roles of multidisciplinary team members, (2) appropriate assessment tools for setting and culture, and (3) multimodal analgesia. Hospitals should have pain teams responsible for pain management that conduct regular audits to ensure that protocols for quality improvement are in place, as well as ensure better patient care. Where such teams cannot be constituted for various reasons, practitioners can be guided by a national protocol based on readily available drugs, and safe alternative strategies.
